# Implementing advance care planning: a qualitative study of community nurses' views and experiences

**DOI:** 10.1186/1472-684X-9-4

**Published:** 2010-04-08

**Authors:** Jane Seymour, Kathryn Almack, Sheila Kennedy

**Affiliations:** 1 School of Nursing, Midwifery and Physiotherapy, University of Nottingham, Queen's Medical Centre, Derby Road, Nottingham, NG7 2UH, UK

## Abstract

**Background:**

Advance care planning (ACP) is a process of discussion about goals of care and a means of setting on record preferences for care of patients who may lose capacity or communication ability in the future. Implementation of ACP is widely promoted by policy makers. This study examined how community palliative care nurses in England understand ACP and their roles within ACP. It sought to identify factors surrounding community nurses' implementation of ACP and nurses' educational needs.

**Methods:**

An action research strategy was employed. 23 community nurses from two cancer networks in England were recruited to 6 focus group discussions and three follow up workshops. Data were analysed using a constant comparison approach.

**Findings:**

Nurses understood ACP to be an important part of practice and to have the potential to be a celebration of good nursing care. Nurses saw their roles in ACP as engaging with patients to elicit care preferences, facilitate family communication and enable a shift of care focus towards palliative care. They perceived challenges to ACP including: timing, how to effect team working in ACP, the policy focus on instructional directives which related poorly to patients' concerns; managing differences in patients' and families' views. Perceived barriers included: lack of resources; lack of public awareness about ACP; difficulties in talking about death. Nurses recommended the following to be included in education programmes: design of realistic scenarios; design of a flow chart; practical advice about communication and documentation; insights into the need for clinical supervision for ACP practice.

**Conclusions:**

Nurses working in the community are centrally involved with patients with palliative care needs who may wish to set on record their views about future care and treatment. This study reveals some important areas for practice and educational development to enhance nurses' use and understanding of ACP.

## Background

Changes in demography mean that those approaching the end of life tend to be older, living in the community and with long term and multiple conditions [1]. It is difficult to clearly identify the transition between 'living' and 'dying' for such individuals and appropriate plans for end-of-life care and transitions to palliative care may be either delayed or never completed, with the resultant outcome that quality of care and experience during dying falls far short of the ideal [2]. 'Advance care planning' (ACP), defined as a process of discussion and review enabling patients to express and, if they wish, to record views, values and specific treatment choices to inform their future care, has been widely promoted as one means of improving care for those living with serious, progressive conditions that are likely to cause incapacity or loss of the ability to communicate wishes to others in the future [3]. ACP commonly results in one or more outcomes [4]. These are, first, instructional or 'advance' directives, often known colloquially as 'living wills', which set on record positive or negative views about specific life prolonging treatments. Those that set out an advance refusal now have legal force in most countries when assessed as valid and applicable. In England and Wales these are called 'advance decisions to refuse treatment' (ADRTs) under the provisions of the Mental Capacity Act [5]. Second, the nomination of an individual to have the authority to represent the patient. One example is the introduction of provisions for 'lasting powers of attorney' for health and welfare under the Mental Capacity Act in England and Wales [5]. A third outcome, which is likely to be applicable to a broad range of patients, involves the setting out of general values and views about care and treatment to inform best interests.

Until recently, most emphasis in policy development internationally has been on the completion of advance directives to enhance precedent autonomy. This trend has been driven in the USA by the implementation of the Patient Self Determination Act during the 1990s [6]. Latterly, emphasis has been placed less on leaving an instruction to guide medical care and more on the potential for ACP discussions to help patients and their families prepare for the last stage of life, review their immediate goals and hopes and strengthen relationships [7-10]. Where ACP is embedded in approaches to changing whole systems of care, it has been found to enable access to palliative care, reduce hospital admissions and interventionist treatment [11,12]. There is some evidence that ACP discussions enable shared decision making in families and satisfaction with decision making [13]. In contrast, there is little evidence that the completion of advance directives alone changes outcomes [12].

In England, the potential for ACP in its broadest sense to contribute to better end-of-life care outcomes has been strongly emphasised in the End of Life Strategy for England [14] and the associated National End of Life Care Programme [15]. The first step of the care pathway set out in the End of Life Strategy is 'discussion as the end of life approaches' involving 'open and honest communication' and 'identifying triggers for discussion'. In the community setting, where most patients spend the majority of their last year of life, there has been a particular emphasis on the elicitation and recording of preferences for place of death, supported by end-of-life initiatives such as the 'Gold Standards Framework' (GSF) [16] which provides a whole systems approach to improving end-of-life care in community settings, and 'Preferred Priorities of Care' (PPC) [17], a tool for recording ACP discussions and any resultant decisions. It is widely acknowledged that community nurses are well placed to engage with ACP because of their pivotal role in provision of primary care based end-of-life care [18,19]. However, the opportunities to engage in ACP promoted by these initiatives have been little studied and there is a lack of understanding of community nurses' professional practice in this area.

The few studies which have examined professionals' (nurses' and doctors') attitudes and experiences show that against a backdrop of largely positive views about the *concept *of ACP, there are worries about the timing, initiation, conduct and recording of ACP discussions and concerns about adequacy of communication skills and the availability of resources [20-22]. The international literature suggests that there are a number of roles that nurses may take in ACP, including providing information and emotional support, facilitating dialogue within families or the health care team, and promoting the completion of advance care records [23,24].

This paper reports on the views, experiences and educational needs in relation to ACP of community nurses working with patients with palliative care needs in England with a view to informing practice and policy in this area. The nurses were participants in a larger community based study exploring end-of-life care concerns and educational needs among older adults and their care providers [unpublished report available on request to the authors]. The specific aims of the aspect of the study reported here were to:

• To examine how community nurses working in palliative care understand ACP and their roles within ACP.

• To identify factors that may facilitate or constrain community nurses' implementation of ACP during patient care.

• To identify community nurses' educational needs to assist them in implementation of ACP practice.

## Methods

The wider study took place between May 2007 and July 2009, with data collected from nurses in 2008. An action research framework underpinned the conduct of the whole project. Action research places emphasis on collaborative working between multiple partners in gaining practical knowledge to effect change. It draws upon different fields of influence including critical thinking and feminism [25], and seeks to produce findings which have direct applicability to the issues being studied. Ethical committee approval was gained through the UK National Research Ethics Service.

To access community based nurses with diverse roles in palliative and end-of-life care we recruited nurses who were affiliated to two Cancer Networks, via local end-of-life facilitators who posted letters to the nurses on our behalf. A meeting was also held for those interested in hearing more about the study, which provided an opportunity for nurses to shape the objectives of this aspect of the study. Nurses indicated interest by either returning a reply slip to the project team or emailing the lead author. We recruited 23 community-based nurses, with diverse roles and levels of experience. Three had qualified between 1970 and 1979; 11 between 1980 and 1989; 7 between 1990 and 1999 and two had qualified since the year 2000. Each of the nurses had received some level of training about ACP although this varied in terms of its depth and content. For most, it had taken the form of attendance at local study days about the Mental Capacity Act or local practice development meetings. Table 1 details the nurses' roles.

**Table 1:** Roles of nurses who took part in focus groups

Clinical nurse specialists in: palliative care (Macmillan nurses), heart failure or respiratory care	9
Hospice nurses	4
Community matrons	4
District nurses	3
Community staff nurse	1
Community psychiatric nurse	1
End-of-life care programme facilitator	1

The nurses took part in 6 focus group discussions about their experiences of providing end-of-life care and views about ACP. We decided to have six focus groups so that each would involve three or four nurses to ensure that nurses had time to talk in some detail about their experiences and views. Three follow up workshops with nurses who had participated in the discussions focused on collaborative interpretation of the focus group data and identification of key themes and developing ideas about educational resources for ACP. An aide memoire was designed and used in the focus group discussions to enable the nurses to reflect on:

• When they had first heard of 'advance care planning'

• Their knowledge and understanding of ACP

• Their views about their contribution and roles in ACP

• Their experiences of implementing ACP practice in patient care

• Their perceptions of challenges or barriers to ACP

• Their training and education needs

The aide memoire was developed in the light of existing literature and following consultation with the nurses at the recruitment meeting.

The focus groups were transcribed with nurses' permission and analyzed with the aid of the qualitative data analysis package NVIVO [26]. We used Strauss and Corbin's [27] constant comparative method to generate categories, patterns and themes from the transcribed textual data relating to experiences and perceptions. The data were initially analyzed by one research team member. Emerging categories and themes were subsequently verified by the research team at a dedicated project meeting and then discussed with the nurses at the follow up workshops. This acted as a form of respondent validation [28] and also generated new insights into the interpretative emphasis we should place on the findings. We do not claim that we have been able to reach data saturation and recommend that further research takes place to check the transferability of the results presented here.

## Results

### First encounters and understandings of ACP

Most of the community nurses had first heard of the term ACP between two and three years prior to the focus group discussions. Nurses identified as sources of information about ACP the new documentation being introduced in practice as a result of the Mental Capacity Act [5], discussions about practice and policy development taking place locally and information related to care planning 'tools' such as the Gold Standards Framework. However, most reported not feeling confident they properly understood the various possible components of ACP:

I think, maybe for me, it was when I worked in (locality) which was over two years ago, we started to go to GSF meetings...over the last two or three years it's been coming in but now a little bit more formally and a little bit more structured I suppose (Community Staff Nurse).

Some perceived that ACP was associated with a very particular set of paperwork and forms, generated by national legislation and policy development, which seemed to imply formalization of everyday practice among individual practitioners. Some recalled being confused about the differences between day-to-day 'care planning', which they regarded as a key aspect of their role, and the more unfamiliar ACP:

I think one of the problems-sort of being on the outside looking in - is that a lot of DNs think, oh not another project, not more paperwork, and it's been in a way perhaps not greeted with huge enthusiasm, although as people have said here before, it's something that a lot of district nurses and healthcare professions say; we've been doing this for, we've done this but we haven't actually formalized it, and that's very much how I see the ACP (Hospice Nurse).

I think, when I first heard about it, it was probably about two/three years ago, I can remember someone talking about it and really thinking what's different about that? And not quite working out exactly what it was; how it differed from ordinary care planning, in other words. And I don't think it was until I got involved, I changed job, and ... got involved with the End-of-life Care Programme, and then obviously it made much more sense. (End-of-Life Care Programme Facilitator).

One Community Matron with management and support responsibilities for other staff recalled her gradual realization, after considerable anxiety, that ACP involved documentation and communication of familiar everyday practice.

I was like 'oh my God what do I need to do, what do I need to do', but we don't need to do anything [different] just document the conversations... we just need to communicate them to other people (Community Matron).

### The contribution of ACP to nursing practice in end-of-life care

Many of the nurses communicated their perceptions of the meaning and potential value of ACP by recalling personal experiences in their family. These personal reflections prompted nurses to identify how, in spite of changes in rhetoric, care at the end of life in their experience tends to be surrounded by a 'curative' culture which forecloses on the possibility of preparation for death and poses a barrier to planning supportive services for dying patients and their families. They perceived the role of the nurse in ACP as an opportunity to shift this emphasis, with ACP seen as an opportunity to celebrate excellent clinical practice:

At its best, it opens up a dialogue which creates a relationship, hopefully a therapeutic relationship, between the clinical person and the patient, and also involving the family if the patient or resident wants the family involved... (End-of-Life Care Programme Facilitator).

The facilitation of 'choice', backed up by resources to enable more than one option for care, was seen as valuable. Similarly, the provision of a framework to enable conversations with patients who wanted to talk about their concerns for the future was viewed to be important. Some nurses reported being more aware as a result of debates about ACP of 'prompts' or 'cues' with which patients may introduce issues about the end of life. For example, one nurse reported how an older person for whom she cared told her:

...I don't need to buy any clothes now; I'm 78 and what I've got in my wardrobe will see me out' (Community Matron).

This nurse described prompts such as these as 'hooks' to hang the next piece of conversation on while attentively following the lead of the patient and thus adapting the pace of the conversation to their degree of comfort with what might otherwise be 'dangerous' territory.

The use of ACP as a means for enabling communication in families was seen as another potentially beneficial factor, providing opportunities for nurses to work with families to build closer relationships and resolve points of conflict or silence:

...you often get families and patients where they're not talking, each is protecting the other, each thinks that they're aware of the reality of the situation and the other person isn't and so it can be useful as it helps to ease dialogue between them and bring them to the same place and the same realisation that ... both parties are aware of the seriousness of the situation and the closeness of the end of life... [it] is very useful to clear the air in some cases [while being] prepared for the fact that you may never get resolution with some people...you might actually create discord (Macmillan Nurse).

It was perceived that where facilitating family communication worked well, the fact that family members became more aware of patients' views and concerns sometimes assisted them subsequently during bereavement. Nurses recognised that this work required attributes of empathy and understanding, as well as knowledge and skills in communication and awareness of the components of ACP.

Some nurses perceived that adopting ACP practices meant that patients' views about some important elements in their care were more likely to be both recorded in their care plan and acted upon, with the result that patients were less likely to be admitted to hospital. In addition, nurses perceived that patients were more likely to continue to express a wish to be cared for at home if preferences that were important to them could be identified and met:

... we're asking, you know, you're asking patients where do you want to be, what's your wishes, you know. (Community Matron).

... a patient we've nursed quite recently with motor neurone disease - he...knew exactly what he wanted. He wasn't going to have a peg feed, he wasn't going to lie down in his bed, he wasn't going to sleep on a pressure reliever mattress, he was going to go upstairs ...we had to really accommodate that.... (District Nurse).

### Challenges and barriers to ACP

Nurses identified a number of challenges and barriers to the effective implementation of ACP, which are summarized in Table 2.

**Table 2:** Challenges and barriers to ACP

*Challenges*	*Barriers*
Identifying the best time and most appropriate person to introduce ACP issues to patients	Lack of resources (including time and end-of-life services) with which to meet patients' preferences and support family carers
Managing differences in staff understandings of ACP in the wider health care team	Lack of public and patients' awareness about ACP and other end-of-life issues
Managing the emphasis on instructional directives and the drive to bureaucratize ACP practice	Taboos and fears about death and dying among public and patients
Documentation and communication of ACP discussions across health care systems	

Managing the potential conflict or difference between patient and family carers' views	

#### Challenges to ACP

Participants highlighted concerns about the timing of ACP and the relationship between their responsibilities towards patients in the ACP process and the responsibilities of other staff:

I found it interesting, on a GSF form in one practice we've got preferred place of death, and often the GPs will say 'oh no, it's too early to talk about that yet' (District Nurse).

But when do they need it? Is it from diagnosis? And I think that's the difficult thing because obviously consultants don't have time to do it, registrars in hospital don't have time to do it, and obviously it comes down to [Macmillan] nurses doesn't it, [or] support nurses within the hospital, because that's usually where the diagnosis is made (Macmillan Nurse).

Participants observed that in their experience GPs are often reluctant to consider and discuss specific decisions relating to ACP with patients or their representatives, whether in the community or in care homes. It was felt that this reluctance arose from discomfort among GPs about raising *any *ACP issues with patients, for fear of raising issues about the end of life 'too soon'.

Nurses with responsibilities for patients with non-cancer long term conditions were especially aware of the issue of timing, given the difficulties of prognostication in the latter and the risk of raising issues about end-of-life care at an inappropriate time that would harm the patient and not be congruent with their coping strategies:

Patients with heart failure and COPD may be living for 10-15 longer years. So I suppose it's pitching just when it's appropriate to have those dialogues, and I think it's different for every person, and I think the same as has been said earlier that there are some people who are going to be very happy, for want of a better word, to discuss that, and there are other patients who don't want to go there (Community Matron).

In all the focus groups, concerns were raised about the bureaucratization of ACP leading to a potentially blunt, harmful 'one size fits all' approach:

... what I have seen unfortunately, is sometimes it's used as more of a checklist, you know, with tick boxes ... (End-of-Life Care Programme Facilitator).

One Macmillan Nurse perceived there was a danger that if nurses and other practitioners were encouraged to regard ACP as a set of procedures or a 'check list of questions' this could effectively subvert the goals of good end-of-life care practice: patient centred care and communication guided by expert clinical judgment.

Nurses also perceived that the wider rhetoric surrounding ACP directed the focus of what practice was in existence towards instructional directives ('advance decisions to refuse treatment'), even if these might be of little relevance to the concerns of most patients. In particular, they perceived that some patients, on admission to hospital were being asked about resuscitation decisions inappropriately and in the absence of any wider discussion about care:

It's interesting though when a patient's taken into hospital now there is a resus status put on them straightaway (Heart Failure Nurse Specialist).

But straightaway they were talking to her daughters about her resus status, you know, that was the first thing that when she got out of the admissions hall that happened ... (District Nurse).

However, in contrast to this, nurses also observed that GPs were often reluctant to engage in discussions about resuscitation or any other end-of-life issues with patients. Nurses perceived a general reluctance to disengage from the 'active' curative mode of care resulted in GPs not acting on the perceptions of nurses or relatives about patients' wishes, even when these had been recorded in an advance care plan. For example, one nurse recalled a patient who she had helped to set out ACP wishes. This included his wish to not go into hospital but to be cared for in his care home at the end of his life:

... a duty doctor was called out in the middle of the night, and they took him into hospital, and unfortunately he died in hospital, which is not what he wanted, [it] caused a lot of issues for his family as well ... And I think the care home staff at the time were pretty adamant his wishes are that he doesn't, but the duty doctor was: 'no he is going', and sort of overruled it all.... (Community Psychiatric Nurse).

Lack of readily available or clear documentary evidence of patients' advance statements and uncertainty about the status of the wishes of close family members in relation to patients' best interests were seen as reasons why medical staff and senior nursing staff might take the least 'risky' course of action when presented with an unfamiliar patient who was acutely ill towards the end of life. One participant recalled the dilemma facing a colleague when dealing with a care home resident whose family expressed a strong preference that he should not be taken into hospital:

(My colleague) was actually put into a bit of dilemma because [patient] was really very ill, and he subsequently died ... she wanted to send him to hospital because he needed hospital treatment. But the daughter had said expressly ... she preferred him to stay in the residential home and got very angry when he was admitted to hospital, but it wasn't recorded anywhere (District Nurse).

Documentation, storage and retrieval of ACP records were perceived as a significant issue across systems of care, especially when patients had many sets of notes and multiple admissions to hospitals.

A further challenge to ACP was the potential conflict between the rhetoric which locates the patient as a free and 'autonomous' agent, with the reality that decisions are made by patients only in the context of relationships with partners or other relatives. A lack of resources to support family carers was perceived as one reason why there might be a disjuncture between patients' and carers' views. A District Nurse outlined an example where a patient wished to be cared for at home but the family were worried about whether they could cope:

... the family were so concerned, worried, although we assured them they'd have a great care package, in reality... it doesn't always come to fruition and there isn't always the care there to support those families... We can't guarantee 24-hour cover but we will try our utmost (District Nurse).

The issue of resources is further examined below.

#### Barriers to ACP

Inadequate resourcing was identified as a key barrier to the implementation of ACP. Nurses perceived that ACP could only be implemented authentically if there were adequate services and resources in place to engage with ACP, to support any choices that patients might record for their future care towards the end of life and provide support to family carers. The nurses below are reflecting on patients' choices for care at home and in a hospice respectively:

... you can try and get the services together and coordinate them, but often they're not there. And I think people can manage very well at home if that's where they want to die as long as we've got the services to keep them at home and to support them (Macmillan Nurse).

Certainly, around heart failure at the minute we do struggle for palliative care support. There isn't a specific unit that patients can go into. When they talk about the hospice, there's actually only day care hospice. X Hospice is only for cancer patients (Heart Failure Nurse Specialist).

A further barrier to ACP perceived by the nurses was a widespread lack of knowledge among the general public, patients and their family members about the availability of help and support during illness and end-of-life care, and a contemporary tendency to not think about one's reaction to serious illness until it actually occurs:

People don't know ... what they want until they're in that situation. Because often people will say to me I didn't know there were all these services out there (Macmillan Nurse).

Nurses also perceived that patients and the public lacked knowledge about the course and outcome of common life-limiting conditions. This created a further barrier to ACP conversations, since many patients perceived they were irrelevant to their situation.

More generally, nurses perceived that patients had many fears about death and illness, which combined to create a taboo surrounding the subject. Fears identified included being frightened of death; fears about going into hospital; about being alone and dying alone. These were all perceived as creating barriers to discussion and yet nurses described how fears could be alleviated once patients were encouraged to put into words what they were most worried about:

And it's also sort of about unpicking why people are ...maybe to facilitate the talk [there is a need] to actually unpick that, what is the fear around, for those people who don't want to talk about it yet (Macmillan Nurse).

### Perceptions about training and education

Among the greatest challenges that nurses perceived to be associated with ACP were their own and colleagues' knowledge and skills about communication practice, recording and follow up:

...we've still got - when you look at teams - a lot of nurses that aren't confident to have those conversations. They say: ' well you like palliative care, you're good at it', and they back off ...That's my worry - the confidence of the staff, teaching them to do it and then following it through (Macmillan Nurse).

I've been in the post three years, so for me it's the uncertainty or where you do document all this information and actually how you can get it through to other people so the patients' wishes are respected - the documentation is a big thing for me (Community Matron).

Nurses recommended that training and education should occur in several ways. Alongside formal training and education, whether by face-to-face teaching or distance learning, some saw the use of mentorship and apprenticeship styles of training as crucial, so that less experienced staff could learn from their more experienced colleagues:

I think there is so much to learn about communication skills and dealing with patients which you can emulate from a role model. And I feel very passionately that junior nurses need to work with senior nurses much more at the bedside, not in the classroom because I think there's a theory and practice divide (Macmillan Nurse).

Those who were involved in care homes perceived a need to provide ongoing support and mentorship of this type to care home staff, particularly to care home managers, so that ACP could gradually become embedded in practice there and so that care staff could gain confidence in dealing with GPs and visiting clinical staff.

Drawing on their experiences of receiving training which had largely focused on the implementation of the Mental Capacity Act, nurses recommended that the following should be included in any education programme to ensure familiarity with the broader aspects of ACP:

• Design of 'real' scenarios for training, which reflected the reality of daily practice and reflected the variety of patients and people encountered during community nursing

• Design of a flow chart to inform nurses and others about the various stages of ACP

• Practical advice about communication and documentation.

Those nurses who were already involved in ACP practice, perceived the importance of ongoing support/clinical supervision as a means of building confidence and safe practice. This was perceived to be just as crucial as knowledge and skills training.

## Discussion

This paper reports one aspect of a larger study, which recruited a relatively small number of community nurses working with patients with palliative care needs in two cancer networks in England. Caution should thus be exercised in generalizing from the findings presented here. We were reliant on nurses being invited to participate in the study by a gate keeper, due to ethical committee requirements, and therefore cannot be certain that every nurse who may have been interested and able to participate received information about the study. In addition, those that took part were self selecting and it is probably the case that they had a particular interest in the topic at hand. Nonetheless, we worked collaboratively with the end-of-life facilitators who invited participants on our behalf to publicize the study and achieved participation from nurses in very diverse roles. Moreover, by holding a preliminary meeting, we were able to gather some opinions from the participants about the key objectives we should address and were subsequently able to involve the participants in a respondent validation exercise and in developing recommendations about the content of educational programmes for ACP. It should be noted that the focus group design may have meant that possible differences between specialist palliative care nurses (who mainly looked after cancer patients) and non specialist community nurses (who looked after cancer patients and many others) were obscured; indeed we found them to have broadly similar views and experiences. An alternative explanation for this similarity is perhaps because ACP is a relatively new concept in England.

The study took place at a time when considerable policy attention in the UK was being directed towards the need to implement ACP practice in the context of a National End of Life Strategy [14], which was about to be published when the data were collected. In addition, a new Mental Capacity Act [5] had recently been introduced with provisions allowing individuals to make legally binding advance decisions to refuse treatment or to nominate a proxy for health and welfare. Together, these created a new set of circumstances for the nurses who were keenly aware of the need to develop their practice accordingly. Although most nurses had little detailed knowledge of ACP in terms of the Mental Capacity Act, they reported that the broader aspects of ACP, such as enabling patients to express their personal preferences for styles of care and developing relationships to facilitate communication, were an integral aspect of their practice. To this extent, ACP was seen as an opportunity to celebrate excellent nursing practice. They described a range of ways in which they used the principles of advance care planning to facilitate dialogue between patients and their families and shift the focus of clinical intervention towards palliative care.

However, concerns were expressed by the nurses that these broader aspects of ACP were at risk of being overshadowed by a disproportionate concern on the part of senior managers with advance decisions to refuse treatment. These were perceived to be overly medically oriented, out of step with the concerns of the majority of patients and somewhat blind to the role of nurses providing palliative care to patients. Similar findings have been found in a study of district nurses' perceptions of their role in palliative care [19] which revealed that district nurses feel that they have a central role in the provision of such care to patients at home which is undervalued and poorly recognized by others.

While community nurses perceived that they have a crucial role in 'opening the door' to ACP with patients they were concerned to time such discussions sensitively, against a cultural backdrop that does not encourage open discussion of death. One aspect to the issue of timing related to a concern that, in addressing ACP issues with patients, nurses risk being out of step with GPs and hospital doctors, whom they perceive are either yet to afford ACP a high priority or do not feel comfortable about raising it until very late in a disease trajectory. Horne et al [20] have described how nurses working to develop ACP practice with patients with lung cancer strove to identify a 'window of opportunity' when ACP issues can be raised with patients but were worried that any development of their practice with such patients may not be complemented by the approach of other staff involved in patient care. A later study of GPs and community nurses found that there was a tendency for both to wait until patients raised issues of relevance to ACP [22]. More research is needed to explore how community nurses and indeed other health care staff initiate ACP discussions.

This study has shown that traditional power differentials between nursing and medicine can pose a barrier to the team working and discussion necessary for the implementation of ACP. A literature review about inter-professional team working in primary and community care has highlighted the need for clear, shared goals to be established to enable effective team working [29]; this is a particularly pertinent issue in managing transitions to palliative care for patients in the community approaching the end of life.

In addition, nurses perceived risks of ACP becoming a bureaucratic 'tick box' exercise as a result of a culture of managerialism with the potential effect perceived of subverting good practice in end-of-life care. A similar and broader trend has been described in a seminal paper about the 'routinisation of hospice' [30]. Avoiding this requires policy makers and clinical managers to appreciate that guidance and protocols for ACP must be subject to professional judgment about their use. This will involve professionals engaging in an ethical analysis of the risk and benefits of ACP for any particular person, using the principles of biomedical ethics (autonomy, beneficence, non- maleficence and justice) [31].

Indeed, nurses perceived a range of moral and ethical concerns to be associated with ACP [32]. In particular, they were concerned that ACP can only be implemented authentically if there are adequate support services to meet patients' preferences, support family caregivers and enable timely and secure communication of records of preferences across systems of care. In addition, the nurses recognized that the practice of ACP could be time consuming; a challenge in the context of an already unpredictable workload [19].

Among the greatest challenges that nurses perceived to be associated with ACP were their own and colleagues' knowledge and skills about communication practice, recording and follow up. A need for careful clinical supervision was perceived, since ACP can raise issues which have the potential to engage with fears and emotions within nurses' biographical lives [33]. The inclusion of ACP issues into communications skills training is important if nurses can fulfill their potential as key players in raising and discussing ACP issues with their patients but must be accompanied at the level of practice by appropriate mechanisms of ongoing support and supervision so that nurses can reflect upon but not be disabled by concerns about illness and death that inevitably surface in ACP work. Not affording formal recognition of the emotional toll of palliative focused work on district nurses has been reported as a barrier to the implementation of palliative care [19].

## Conclusions

Community nurses have a key role in providing palliative care to patients in the community and are well placed to facilitate a process of ACP which has the potential to improve the quality of end-of-life care that patients receive. This paper has highlighted some critical areas of concern if this potential is to be fully realized.

## Competing interests

The authors declare that they have no competing interests.

## Authors' contributions

JS conceived and led the study, participated in data analysis and wrote the first draft of this paper. KA and SK assisted in the conduct of the study, participated in data analysis and edited the paper. All authors read and approved the final manuscript.

## Pre-publication history

The pre-publication history for this paper can be accessed here:

http://www.biomedcentral.com/1472-684X/9/4/prepub
